# Antimicrobial resistance of bacterial pathogens isolated from the infections of post maxillofacial surgery

**DOI:** 10.25122/jml-2021-0149

**Published:** 2022-08

**Authors:** Zaid Mustafa Akram, Khalid Burhan Khalid, Qaisar Khaleel Oraibi, Maadh Fawzi Nassar

**Affiliations:** 1 Department of Dentistry, Al-Rafidain University College, Baghdad, Iraq; 2 Department of Dentistry, Dijlah University College, Baghdad, Iraq; 3 Department of Dentistry, Al-Esraa University College, Baghdad, lraq; 4 Department of Chemistry, Faculty of Science, University Putra Malaysia, Selangor, Malaysia

**Keywords:** maxillofacial infections, prevalence, bacteria, antimicrobial resistance

## Abstract

Inappropriate antibiotic prescriptions contributed to a global issue of antimicrobial resistance. This study aimed to assess the prevalence of bacterial pathogens and antimicrobial resistance isolated from maxillofacial infections (MIs). Two hundred and twenty-two patients with different MIs were included in this study. Swab samples were taken from the site of infections. Samples were cultured, and isolated bacteria were identified using various biochemical tests. Antimicrobial resistance patterns of isolates were assessed by the disk diffusion method. The mean age of the patients was 50.8 years. The male-to-female ratio was 127/95 (P<0.05). Smoking and alcohol consumption were found in 60.36% and 37.38% of patients, respectively. Most patients had a ≤1-week infection duration (P<0.05). Abscess lesion was the most predominant infection type (P<0.05). The prevalence of aerobic bacteria among abscess, pus localization, and deep facial infections was 59.33%, 64.28%, and 46.66%, respectively. The prevalence of anaerobic bacteria among abscess, pus localization, and deep facial infections was 40.66%, 23.80%, and 53.33%, respectively. *Staphylococcus aureus* (10.36%) and *Prevotella buccalis* (8.55%) had the uppermost distribution amongst all examined samples. Isolated bacteria exhibited the uppermost resistance rate toward penicillin (65.76%), tetracycline (61.26%), gentamicin (58.10%), and ampicillin (57.65%) antimicrobials. The lowest resistance rate was obtained for linezolid (25.67%), ceftriaxone (31.08%), and azithromycin (31.08%) antimicrobials. Linezolid, ceftriaxone, and azithromycin had effective antimicrobial activities toward bacteria isolated from MIs. Therefore, cautious antibiotic prescription might decrease the prevalence of antimicrobial resistance in dental and maxillofacial infections.

## INTRODUCTION

Maxillofacial infections (MIs) are commonly attributed to the face and oral cavity [1]. Given the important anatomical position of the maxillofacial region, infections of this part may expand to other sites, including the respiratory system, brain, and mediastinum, and subsequent septicemia and even death may occur [2]. MIs are primarily self-limiting and can be treated quickly. However, there is a risk of death from airway obstruction and even infection spread [3, 4].

Bacteria are considered the most frequent causes of MIs globally [5]. Numerous investigations described that the *Streptococcus species (spp.), Staphylococcus aureus (S. aureus), Klebsiella pneumonia (K. pneumonia), Corynebacterium spp*., *Pseudomonas aeruginosa* (*P. aeruginosa*), *Enterobacter aerogenes (E. aerogenes), Enterococcus faecalis (E. faecalis), Haemophilus spp*., *Aggregatibacter actinomycetemcomitans (A. actinomycetemcomitans)*, and *Acinetobacter baumannii (A. baumannii)* are the most frequent aerobic bacteria responsible for MIs [6–10]. Additionally, *Prevotella spp*., *Peptostreptococcus spp*., *Bacteroides forsythus (B. forsythus), Fusobacterium nucleatum (F. nucleatum), Porphyromonas gingivalis (P. gingivalis), Veillonella spp*., and *Eubacterium spp*. are measured as the significant anaerobic bacteria isolated from MI cases [7, 10].

Treatment of most MI cases requires an antimicrobial prescription. However, most aerobic and anaerobic bacteria responsible for MI exhibit a high resistance rate toward common antimicrobials [11]. The high antibiotic resistance rate of aerobic and anaerobic bacteria responsible for MI cases has been reported toward aminoglycosides, tetracyclines, penicillins, cephalosporins, quinolones, and other important classes of antimicrobials [12–14]. Thus, assessing MIs etiologic agent antimicrobial resistance can help identify the best antibiotics to treat and control the infection.

Given the high importance of MIs as common and complicated bacterial infections with the emergence of antimicrobial resistance, existing research was conducted to assess the prevalence and antimicrobial resistance of aerobic and anaerobic bacteria isolated from different types of MIs.

## Material and methods

### Study population, inclusion, and exclusion criteria

A total of 300 patients were included in the study from October 2019 to October 2020.

Inclusion criteria: patients with bacterial infections of odontogenic origin, including dentoalveolar abscess, those with deep fascial space spreading infections, and others with infections causing localization of pus in the head and neck, were included in the study.

Exclusion criteria: patients with viral and fungal infections, infected cysts, neoplastic lesions, and those without known infections were excluded from the study. Additionally, patients with antibiotic therapy (over the past 30 days) and who used antiseptic mouth rinses (over the past 24h) were excluded from the survey. Pregnant women, patients with liver, gastrointestinal, and kidney disease, and those with positive Covid-19 and HIV tests were also excluded.

### Samples

Aspiration sites were cleaned with alcohol (Merck, Germany). Saliva was continuously aspirated during the sampling. A separate sterile needle was used for pus aspiration from each patient. If aspiration was unsuccessful, a separate sterile swab was used for pus or exudate collection. Samples were transferred to the laboratory using the thioglycollate broth (Merck, Germany) media. Geographical information of the targeted population was recorded accurately.

### Bacterial isolation and identification

All samples were separately cultured on the blood agar media (Merck, Germany) for aerobic incubation, chocolate agar (Merck, Germany) for microaerophilic incubation, and anaerobic blood agar (Merck, Germany) for anaerobic incubation. The blood agar media was prepared using the blood agar base (Oxoid, UK) with 5% defibrinated sheep blood. The anaerobic blood agar media was prepared using the fastidious anaerobe agar (Oxoid, UK) with 5% defibrinated sheep blood. All media were incubated at 37ºC. All isolates were subjected to Gram-staining. Isolates grown on the blood agar and chocolate agar were Gram-stained after 24h of growth in air and CO_2_, respectively. Isolates grown on the anaerobic blood agar were Gram-stained after 48h. Gram-negative and Gram-positive bacteria were tested using various biochemical tests according to the Analytical Profile Index (API) system. Gram-negative bacillus bacteria were identified using the API 20E [15]. The catalase production test was used for Gram-positive coccoid bacteria. All catalase-negative bacteria were tested for the hemolytic reaction, and growth in the media contained 6.5% NaCl. Catalase-positive bacteria were tested for coagulase production, resistance to Novobiocin, and growth on the mannitol salt agar (MSA, Merck, Germany). Anaerobic bacteria were identified using the AP120A procedures [16]. Anaerobic culture was provided using the anaerobic jar (Oxoid, UK) and MART system (Lichtenvoorde, The Netherlands, 80% N_2_, 10% O_2_, and 10% CO_2_) [17–19].

### Antimicrobial resistance properties

Procedures introduced by the Clinical and Laboratory Standards Institute (CLSI) [17] were applied for this goal. For this purpose, 0.5 McFarland turbidity standard was provided using an optical density comparable to the density of a bacterial suspension with 1.5x10^8^ colony forming units (CFU/ml). Mueller-Hinton agar (Merck, Germany) was used for bacterial culture. Diverse antimicrobial disks, including penicillin (10 µg/disk), ampicillin (10 µg/disk), amoxicillin (25 µg/disk), levofloxacin (5 µg/disk), ceftriaxone (30 µg/disk), clindamycin (2 µg/disk), vancomycin (30 µg/disk), azithromycin (15 µg/disk), erythromycin (15 µg/disk), metronidazole (5 µg/disk), gentamicin (10 µg/disk), linezolid (30 µg/disk), and tetracycline (30 µg/disk), were placed on media. Microbial media with placed disks were incubated (35°C for 24h). Aerobic and anaerobic conditions were applied according to the targeted bacteria. CLSI guidelines were applied for susceptibility analysis [20–24]. All antibiotics were selected according to the CLSI and the frequency of usage to treat oral infections.

### Numerical evaluation

Data collected from the experiment were analyzed using the SPSS/21.0 software (SPSS Inc., Chicago, IL) [25–28]. Qualitative data were examined using the Chi-square test and Fisher's exact two-tailed test. A P-value less than 0.05 was determined as a significance level [29–31].

## Results

### Demographic characteristics

The demographic characteristics of the examined population are shown in Table 1. Of 300 patients examined in this survey, 222 (74%) were included in the study. The mean age of the studied population was 50.8 years. The male-to-female ratio of the examined population was 127/95 (P<0.05). Histories of smoking and alcohol consumption amongst patients were 60.36% and 37.38%, respectively. One-hundred and thirty-nine out of 222 (62.61%) patients had a ≤1-week infection duration (P<0.05). Most of the examined patients had an abscess (67.56%) (P<0.05).

**Table 1:** Demographic characteristics of the study population.

Demographic characteristics		Study population (n=222)
**Mean age (SD) (year)**		50.8 (15.2)
**Sex (M/F)**		127/95
**Mean weight (SD)**		67.3 (17.6)
**Smoking (%)**		60.36
**Alcohol (%)**		41.89
**Duration of infection (%)**	**≤1 week**	139 (62.61)
	**>1 week**	83 (37.38)
**Clinical findings**		
**Abscess (%)**		150 (67.56)
**Pus localization (%)**		42 (18.91)
**Deep fascial infections (%)**		30 (13.51)

### Prevalence of bacteria

Table 2 shows the distribution of bacteria amongst studied samples of maxillofacial infections. Two kinds of bacteria, aerobic and anaerobic, were isolated from the examined samples of maxillofacial infections. The prevalence of aerobic bacteria amongst all studied samples was 58.55%. The prevalence of aerobic bacteria among samples collected from the abscess, pus localization, and deep facial infections was 59.33%, 64.28%, and 46.66%, respectively. The prevalence of anaerobic bacteria amongst all studied samples was 41.44%. The total prevalence of anaerobic bacteria among samples collected from the abscess, pus localization, and deep facial infections was 40.66%, 23.80%, and 53.33%, respectively. The prevalence of aerobic and anaerobic bacteria amongst examined samples was statistically significant (P<0.05). *S. aureus* (10.36%) and *P. buccalis* (8.55%) had the uppermost distribution amongst all examined maxillofacial infections. *S. aureus* (10%) and *K. pneumonia* (6.66%) aerobic bacteria had a higher distribution among the abscess samples. *P. buccalis* (8.66%) and *Peptostreptococcus spp*. (7.33%) anaerobic bacteria had a higher distribution among the abscess samples. *S. aureus* (9.52%) and *S. pyogenes* (9.52%) aerobic bacteria had a higher distribution among the pus localization samples. *P. buccalis* (7.14%) and *Peptostreptococcus spp*. (7.14%) anaerobic bacteria had a higher distribution among the pus localization samples. *S. aureus* (13.33%) and *S. pyogenes* (10%) aerobic bacteria had a higher distribution among the deep facial infectious samples. *P. buccalis* (10%) and *P. gingivalis* (10%) anaerobic bacteria had a higher distribution among the deep facial infectious samples. Statistically, a significant difference was obtained between the types of samples and the prevalence of bacteria (P<0.05).

**Table 2:** Distribution of bacteria among maxillofacial infections samples.

Bacterial species			Distribution (%)			
			Abscess (150 samples)	Pus localization (42 samples)	Deep facial infections (30 samples)	Total (222 samples)
**Aerobic**	*Streptococcus*	*S. viridans*	8 (5.33)	3 (7.14)	2 (6.66)	13 (5.85)
		*S. mutans*	4 (2.66)	1 (2.38)	1 (3.33)	6 (2.70)
		*S. pneumoniae*	2 (1.33)	1 (2.38)	-	3 (1.35)
		*S. oralis*	3 (2.00)	2 (4.76)	-	5 (2.25)
		*S. mitis*	3 (2.00)	-	1 (3.33)	4 (1.80)
		*S. pyogenes*	8 (5.33)	4 (9.52)	3 (10)	15 (6.75)
	*S. aureus*		15 (10%)	4 (9.52)	4 (13.33)	23 (10.36)
	*K. pneumonia*		10 (6.66)	3 (7.14)	1 (3.33)	14 (6.30)
	*Corynebacterium spp*.		8 (5.33)	2 (4.76)	-	10 (4.50)
	*P. aeruginosa*		5 (3.33)	2 (4.76)	-	7 (3.15)
	*E. aerogenes*		4 (2.66)	1 (2.38)	1 (3.33)	6 (2.70)
	*E. faecalis*		5 (3.33)	2 (4.76)	1 (3.33)	8 (3.60)
	*Haemophilus spp*.		2 (1.33)	-	-	2 (0.90)
	*A. actinomycetemcomitans*		3 (2.00)	-	-	3 (1.35)
	*A. baumannii*		9 (6.00)	2 (4.76)	-	11 (4.95)
	**Total**		**89 (59.33)**	**27 (64.28)**	**14 (46.66)**	**130 (58.55)**
**Anaerobic**	*Prevotella*	*P. buccalis*	13 (8.66)	3 (7.14)	3 (10)	19 (8.55)
		*P. dentalis*	8 (5.33)	1 (2.38)	1 (3.33)	10 (4.50)
		*P. intermedia*	7 (4.66)	2 (4.76)	2 (6.66)	11 (4.95)
	*Peptostreptococcus spp*.		11 (7.33)	3 (7.14)	2 (6.66)	16 (7.20)
	*B. forsythus*		3 (2.00)	1 (2.38)	1 (3.33)	5 (2.25)
	*F. nucleatum*		5 (3.33)	1 (2.38)	2 (6.66)	8 (3.60)
	*P. gingivalis*		10 (6.66)	2 (4.76)	3 (10)	15 (6.75)
	*Veillonella spp*.		2 (1.33)	1 (2.38)	1 (3.33)	4 (1.80)
	*Eubacterium spp*.		2 (1.33)	1 (2.38)	1 (3.33)	4 (1.80)
	**Total**		**61 (40.66)**	**15 (23.80)**	**16 (53.33)**	**92 (41.44)**

### Antibiotic resistance of bacteria

Table 3 displays the antimicrobial resistance pattern of bacterial strains isolated from maxillofacial infections. Isolated bacteria exhibited the highest resistance rate toward penicillin (65.76%), tetracycline (61.26%), gentamicin (58.10%), and ampicillin (57.65%) antimicrobials. The lowest resistance rate was obtained for linezolid (25.67%), ceftriaxone (31.08%), and azithromycin (31.08%) antimicrobials. *S. aureus* isolates exhibited the highest resistance rate toward most examined antimicrobials. Anaerobic bacteria exhibited a higher resistance rate toward metronidazole. Statistically, a significant difference was obtained between the types of bacteria and antibiotic resistance rate (P<0.05).

**Table 3:** Antimicrobial resistance pattern of bacterial strains isolated from maxillofacial infections.

Bacterial species (Numbers)	N. isolates harbored resistance against each antimicrobial disk (%)												
	P10	A10	A25	L5	Cf30	Cln	V30	Az	Ert	Met	G10	Lin	Tet
**S. viridans (13)**	10 (76.92)	9 (69.23)	8 (61.53)	6 (46.15)	4 (30.76)	5 (38.46)	4 (30.76)	4 (30.76)	6 (46.15)	5 (38.46)	9 (69.23)	3 (23.07)	10 (76.92)
**S. mutans (6)**	4 (66.66)	3 (50)	3 (50)	2 (33.33)	1 (16.66)	2 (33.33)	2 (33.33)	1 (16.66)	3 (50)	3 (50)	4 (66.66)	2 (33.33)	4 (66.66)
**S. pneumoniae (3)**	3 (100)	2 (66.66)	2 (66.66)	1 (33.33)	1 (33.33)	1 (33.33)	1 (33.33)	1 (33.33)	1 (33.33)	1 (33.33)	3 (100)	1 (33.33)	3 (100)
**S. oralis (5)**	3 (60)	3 (60)	2 (40)	2 (40)	2 (40)	2 (40)	2 (40)	2 (40)	2 (40)	2 (40)	3 (60)	2 (40)	3 (60)
**S. mitis (4)**	2 (50)	2 (50)	2 (50)	1 (25)	1 (25)	1 (25)	1 (25)	1 (25)	2 (50)	1 (25)	2 (50)	1 (25)	2 (50)
**S. pyogenes (15)**	13 (86.66)	11 (73.33)	9 (60)	6 (40)	7 (46.66)	6 (40)	5 (33.33)	7 (46.66)	10 (66.66)	6 (40)	10 (66.66)	4 (26.66)	13 (86.66)
**S. aureus (23)**	21 (91.30)	20 (86.95)	18 (78.26)	15 (65.21)	10 (43.47)	12 (52.17)	7 (30.43)	10 (43.47)	15 (65.21)	8 (34.78)	17 (73.91)	6 (26.08)	21 (91.30)
**K. pneumonia (14)**	11 (78.57)	10 (71.42)	8 (57.14)	6 (42.85)	4 (28.57)	4 (28.57)	3 (21.42)	4 (28.57)	7 (50)	4 (28.57)	8 (57.14)	3 (21.42)	11 (78.57)
**Corynebacterium spp. (10)**	7 (70)	6 (60)	5 (50)	4 (40)	3 (30)	3 (30)	3 (30)	3 (30)	6 (60)	4 (40)	7 (70)	3 (30)	7 (70)
**P. aeruginosa (7)**	5 (71.42)	4 (57.14)	3 (42.85)	2 (28.57)	2 (28.57)	2 (28.57)	2 (28.57)	2 (28.57)	3 (42.85)	2 (28.57)	6 (85.71)	2 (28.57)	5 (71.42)
**E. aerogenes (6)**	3 (50)	3 (50)	2 (33.33)	2 (33.33)	2 (33.33)	2 (33.33)	2 (33.33)	2 (33.33)	2 (33.33)	3 (50)	3 (50)	2 (33.33)	3 (50)
**E. faecalis (8)**	6 (75)	5 (62.5)	4 (50)	3 (37.50)	4 (50)	3 (37.50)	3 (37.50)	4 (50)	3 (37.50)	3 (37.50)	5 (62.5)	3 (37.50)	6 (75)
**Haemophilus (2)**	1 (50)	1 (50)	1 (50)	1 (50)	1 (50)	1 (50)	1 (50)	1 (50)	1 (50)	-	1 (50)	1 (50)	1 (50)
**A. actinomycetemcomitans (3)**	1 (33.33)	1 (33.33)	1 (33.33)	1 (33.33)	1 (33.33)	1 (33.33)	1 (33.33)	1 (33.33)	1 (33.33)	1 (33.33)	2 (66.66)	1 (33.33)	1 (33.33)
**A. baumannii (11)**	9 (81.81)	7 (63.63)	6 (54.54)	5 (45.45)	3 (27.27)	4 (36.36)	3 (27.27)	3 (27.27)	7 (63.63)	3 (27.27)	8 (72.72)	3 (27.27)	9 (81.81)
**P. buccalis (19)**	11 (57.89)	10 (52.63)	8 (42.10)	6 (31.57)	5 (26.31)	4 (21.05)	4 (21.05)	5 (26.31)	8 (42.10)	13 (68.42)	10 (52.63)	4 (21.05)	11 (57.89)
**P. dentalis (10)**	4 (40)	4 (40)	3 (30)	3 (30)	2 (20)	2 (20)	2 (20)	2 (20)	3 (30)	5 (50)	3 (30)	2 (20)	4 (40)
**P. intermedia (11)**	4 (36.36)	3 (27.27)	3 (27.27)	3 (27.27)	2 (18.18)	3 (27.27)	3 (27.27)	2 (18.18)	3 (27.27)	6 (54.54)	4 (36.36)	3 (27.27)	4 (36.36)
**Peptostreptococcus (16)**	12 (75)	10 (62.5)	8 (50)	6 (37.5)	4 (25)	5 (31.25)	4 (25)	4 (25)	7 (43.75)	12 (75)	10 (62.5)	3 (18.75)	12 (75)
**B. forsythus (5)**	3 (60)	2 (40)	2 (40)	2 (40)	2 (40)	2 (40)	2 (40)	2 (40)	2 (40)	3 (60)	2 (40)	2 (40)	3 (60)
**F. nucleatum (8)**	3 (37.5)	3 (37.5)	3 (37.5)	3 (37.5)	2 (25)	3 (37.5)	2 (25)	2 (25)	3 (37.5)	4 (50)	3 (37.5)	2 (25)	3 (37.5)
**P. gingivalis (15)**	6 (40)	5 (33.33)	4 (26.66)	3 (20)	3 (20)	2 (13.33)	1 (6.66)	3 (20)	3 (20)	7 (46.66)	5 (33.33)	1 (6.66)	6 (40)
**Veillonella (4)**	2 (50)	2 (50)	2 (50)	2 (50)	2 (50)	2 (50)	2 (50)	2 (50)	2 (50)	2 (50)	2 (50)	2 (50)	2 (50)
**Eubacterium (4)**	2 (50)	2 (50)	2 (50)	1 (25)	1 (25)	1 (25)	1 (25)	1 (25)	2 (50)	2 (50)	2 (50)	1 (25)	2 (50)
**Total (222)**	146 (65.76)	128 (57.65)	109 (49.09)	86 (38.73)	69 (31.08)	73 (32.88)	71 (31.98)	69 (31.08)	102 (45.94)	100 (45.04)	129 (58.10)	57 (25.67)	136 (61.26)

P10 – penicillin (10 μg/disk); A10 – ampicillin (10 μg/disk); A25 – amoxicillin (25 μg/disk); l5 – levofloxacin (5 μg/disk); Cf30 – ceftriaxone (30 μg/disk); Cln – clindamycin (2 μg/disk); V30 – vancomycin (30 μg/disk); Az – azithromycin (15 μg/disk); Ert – erythromycin (15 μg/disk); Met – metronidazole (5 μg/disk); G10 – gentamicin (10 μg/disk); Lin – linezolid (30 μg/disk); Tet – tetracycline (30 μg/disk).

## Discussion

Medical sciences have developed in recent years [32]. However, infections remain health-threatening issues [33–35]. The existing research was performed to evaluate the prevalence and antimicrobial resistance of bacterial strains isolated from MI infections. A total of 24 different bacteria were isolated from MI samples. Isolates antimicrobial resistance was tested for 13 different antimicrobials. Findings revealed a higher distribution of aerobic bacteria than anaerobic, particularly in abscess and pus localization samples. Reversely, a higher prevalence of anaerobic bacteria was reported in deep infections. Maybe the anatomical location of infections (superficial or deep) intervenes in the presence of aerobic or anaerobic bacteria. Similarly, a high prevalence of aerobic bacteria was recognized in previous surveys [36]. Among aerobic bacteria, *S. aureus, S. pyogenes, S. viridans*, and *K. pneumonia* had a higher prevalence rate. Kityamuwesi *et al*. (2015) [37] stated that *S. aureus* (23.50%) and *S. viridans* (19.40%) were the most frequently identified aerobic bacteria amongst odontogenic infections in Uganda. Similar patterns of aerobic bacteria were reported in Austria [38], Taiwan [39], Japan [40], and Denmark [41]. Among anaerobic bacteria, *P. buccalis, Peptostreptococcus spp*., and *P. gingivalis* had a higher prevalence rate. Nóbrega *et al*. (2016) [42] stated that Prevotella, *Peptostreptococcus spp*., and *P. gingivalis* were the most frequently identified anaerobic bacteria amongst endodontic infections in Brazil. Additionally, a similar profile of anaerobic bacteria was reported in Japan [43], the United Kingdom [44], and the United States [45]. Kabanova (2017) [46] stated that *S. viridans, S. pneumoniae, S. aureus, K. pneumonia*e, *A. baumannii, E. faecalis*, and *P. aeruginosa* were the predominant bacteria among orofacial infections in Belarus. Brescó-Salinas *et al*. (2006) [47] stated that the prevalence of *S. oralis, E. fecalis, B. forsythus, F. nucleatum, P. gingivalis, P. intermedia*, and *A. actinomycetemcomitans* amongst the odontogenic infections in Spain was 72%, 28%, 28%, 21.80%, 10.90%, and 7.20%, respectively. A Lithuanian survey [48] described that *S. haemolyticus, Bacteroides spp*., and *S. epidermidis* were the predominant bacteria amid the maxillofacial infections. The prevalence of *S. viridans, S. aureus, K. pneumonia*e, *Enterobacter spp*., *S. epididymis, Pseudomonas spp*., *S. mitis*, and *S. oralis* amid the orofacial infections in South Africa were 36.10%, 21.30%, 5.70%, 4.10%, 2.50%, 2.50%, 2.50%, and 2.50%, respectively [49]. A survey conducted in Nigeria [50] reported that the *S. aureus* (22%), *E. faecalis* (2%), alpha-hemolytic Streptococci (4%), *A. baumannii* (2%), *E. aerogenes* (2%), *K. pneumonia*e (16%), *P. aeruginosa* (2%), *P. anaerebius* (4%), *P. denticola* (4%), and *P. intermedia* (4%) were predominant amid the odontogenic infections. The deviation of bacterial culture may be due to the different lifestyles, behaviors, and diets of examined patients in diverse research.

Isolates displayed a high antibiotic resistance rate toward penicillin, tetracycline, gentamicin, and ampicillin antimicrobials. Unauthorized prescription of antimicrobials, self-treatment with antimicrobials, and indiscriminate use of disinfectants are likely explanations for the prevalence of antimicrobial resistance in the present survey. Linezolid, ceftriaxone, and azithromycin prescription may cause better therapeutic effects on maxillofacial infections. Similarly, a high resistance rate toward penicillin, tetracycline, gentamicin, and ampicillin antimicrobials was reported in the United States [51], Australia [52], and the United Kingdom [53]. Kong and Kim (2019) [54] stated that the *S. aureus, S. viridans, K. pneumonia*e, and *E. fecalis* bacteria displayed the uppermost resistance rate against ampicillin, ciprofloxacin, clindamycin, erythromycin, gentamicin, penicillin, and tetracycline antimicrobials [55]. Habib *et al*. (2019) [56] stated that *Staphylococcus spp*., *Streptococcus spp*., and *Klebsiella spp*. isolates of odontogenic infections had a high resistance toward amoxicillin and metronidazole (80–100%). A Chinese survey [57] described boosting resistance rate toward ampicillin (100%) and penicillin (100%) antimicrobials. Possible reasons for antibiotic resistance differences reported in various studies include differences in antibiotic availability, antibiotic prices, over-the-counter antibiotic sales, and antibiotic prescribing procedures. Precise prescriptions based on laboratory results can diminish the risk of antimicrobial resistance among maxillofacial pathogens.

There is no determined document about the exact origin of isolated bacteria. However, the role of food as a vector for these bacteria and also changes in the microflora of the oral cavity are more prone than other reasons [58, 59]. We suggest other authors assess the originality of oral infections and the full genome sequencing of bacterial isolates to assess their genetic similarity.

## Conclusions

The main achievement of this report was the assessment of antimicrobial resistance of bacteria isolated from infections of post maxillofacial surgery in order to identify the best treatment option and the main distribution of bacterial pathogens in these areas. In conclusion, *S. aureus, S. pyogenes, S. viridans, K. pneumonia, P. buccalis, Peptostreptococcus spp*., and *P. gingivalis* were the predominant causes of maxillofacial infections in Iraq. Rendering the disk diffusion findings, linezolid, ceftriaxone, and azithromycin prescription may cause better results in treating maxillofacial infections. Establishing preventive rules in prescribing antibiotics and accurately identifying the main causes of infection in these areas can prevent the spread of antibiotic-resistant strains in post maxillofacial surgery infections. However, several multifactorial surveys should be performed to address more aspects of the antimicrobial resistance bacteria in MIs.

## Personal thanks

The authors would like to thank the Department of Dentistry, Al-Rafidain University College, Baghdad, Iraq, for the support.
